# Maternal-Fetal Circadian Communication During Pregnancy

**DOI:** 10.3389/fendo.2020.00198

**Published:** 2020-04-15

**Authors:** Keenan Bates, Erik D. Herzog

**Affiliations:** Department of Biology, Washington University, St. Louis, MO, United States

**Keywords:** daily rhythm, parturition, melatonin, glucocorticoid, dopamine

## Abstract

This article reviews evidence for maternal-fetal communication about the time of day. We explore the hypothesis that key maternal hormones synchronize daily rhythms in the fetus to regulate gestation duration. These findings may help to predict and prevent preterm birth.

## Introduction

Circadian rhythms in gene expression and hormones are ubiquitous across species and across cell types (1, 2). Familiar examples include daily rhythms in sleep and wake, the nightly rise in melatonin secretion seen in most vertebrates, and the Nobel-prize winning discoveries in fruit flies showing how the *Period* gene negatively regulates its own transcription by repressing the transcription factors, CLOCK and BMAL1, on a daily basis (3, 4). To be useful, these daily rhythms in cells must be synchronized within the body and to the local light-dark cycle. This review focuses on how signals during pregnancy can synchronize daily rhythms between the mother and the fetus.

Daily rhythms depend on clock genes. A transcriptional-translational negative feedback loop generates the near-24 h cycles in most mammalian cell types. BMAL1 and CLOCK, two basic helix-loop-helix-PAS domain transcription factors, heterodimerize, and initiate the transcription of the *Period* (*Per1, Per2*, and *Per3*) and *Cryptochrome* (*Cry1* and *Cry2*) genes (4). The PER and CRY proteins heterodimerize and translocate into the nucleus where they inhibit their own transcription by repressing the activity of BMAL1 and CLOCK (4). As transcript levels decline, the PER and CRY proteins degrade, so that BMAL1 and CLOCK can bind together to activate transcription again (4). Our understanding of the molecular mechanisms underlying circadian rhythms in processes including gene expression, hormone secretion, and their disruption in disease have been reviewed eloquently elsewhere (4–7).

Although circadian rhythms have been studied heavily in a wide variety of organisms, there has been substantial bias to examine chronobiology in males. For example, nearly all studies of the hypothalamic suprachiasmatic nucleus (SCN), a master circadian pacemaker in vertebrates, have been done in males despite early reports of its sexual dimorphism and its role in female endocrinology in humans and other mammals (8–13). Mammalian pregnancy presents a unique situation to study the importance of female circadian biology with daily rhythms in the mother and, later in the pregnancy, rhythms in the fetus which synchronize to each other and to local time (14–17).

## Criteria for Candidate Maternal-Fetal Circadian Coupling Factors

Over the course of pregnancy, the hormonal profile changes to adapt to the needs of the developing fetus (see Table 1). Changes in some of these hormones (e.g., prolactin derived from the mother or lactogens from the fetal placenta) coincide with critical developmental events. Here, we seek to identify maternal signals that can synchronize fetal circadian rhythms and vice versa. We focus on hormonal signals which are strong candidates for communicating time of day information. We limit our discussion to factors that fulfill these five criteria:

1. Humoral factors,
2. derived either from the mother or the fetus,
3. produced in a circadian pattern during pregnancy,
4. with complimentary receptor distributions, and
5. capable of crossing the placental barrier.

**Table 1:** Maternal hormone concentration and peak concentration time in each trimester and in non-pregnant women.

**Hormone**	**Peak time**				**Daytime/Nighttime concentrations**			
	**NP**	**1T**	**2T**	**3T**	**NP**	**1T**	**2T**	**3T**
Melatonin	Follicular and Luteal: 01:00 (18)	00:00–4:00 (9–13 weeks) (19)	00:00–04:00 (24–37 weeks) (19)		41.7 pmol/L (11:00) (19)/ ~200 pmol/L (03:00) (20)	29.97 pmol/L (11:00) (19)/ 245.1 pmol/L (04 :00) (19)	39.1 pmol/L (11:00) (19)/ 663.5 pmol/L (04 :00) (19)	76.5 pmol/L (11:00) (19)/ 663.5 pmol/L (04 :00) (19)
Dopamine	08:00 (Men) (21)	nd	nd	nd	nd	nd	nd	nd
Cortisol	07:00 (22)	07:00–09:00 (23)	07:00–09:00 (23)	07:00–09:00 (23) 08:00 (30–39 weeks) (24)	292 nmol/L (07:00) (22)/ 70 nmol/L (01:00) (22)	149 ng/mL (08:00) (23)/ nd	352 ng/mL (08:00) (23)/ nd	~400 ng/mL (08:00) (23, 24)/ ~200 ng/mL (00:30) (24)
Progesterone	Follicular:11:00 (18) Luteal:14:00 (18)	24:00–01:00 (6–10 weeks) (25)	nd	nd	nd	40.2 ng/mL (24:00) (25)/26.4 ng/mL (08:00) (25)	nd	nd
Estrogen	Follicular: 01:00 (18) Luteal: 02:00 (18) (estradiol)	nd	nd	01:30–03:30 (30–31 weeks) (24) 22:30–00:30 (34–35 weeks) (24) (Estriol)	nd	nd	nd	~6.0 ng/mL 01:30–03:30 (30–31 weeks) 22:30–00:30 (34–35 weeks) (24)/5 ng/mL 09:00–10:30 (30–31 weeks) 4.7 ng/mL 10:00–11:30 (34–35 weeks) (24)

## *Relevant reference for peak time and concentration in parentheses*.

*NP, Non-pregnant; 1T, First Trimester; 2T, Second Trimester; 3T, Third Trimester*.

*Nd, No data*.

*Peak time: Time of day of sample collection and (gestational age of measurements)*.

*Day/Night: concentrations and (time of day of sample collection)*.

## Maternal Circadian Rhythms Entrain Rhythms in the Fetus

The maternal SCN has an important role in providing circadian entraining signals to the fetus. Reppert and Schwartz (14) provided the first evidence for *in utero* daily rhythms when they showed that the fetal rat SCN had higher glucose utilization during the day than at night beginning on embryonic day 19 (E19), 3 days prior to birth (14, 26). When the dam underwent a 12-h shift in the light-dark schedule, the rhythm in glucose utilization in the fetal SCN exhibited a matching phase shift (14). Blinding the mother prior to altering the lighting schedule prevented a phase shift in the mother and the fetus (14). Additionally, lesioning the maternal SCN or knocking out the maternal core clock genes, *Per1* and *Per2*, prevented entrainment of the fetal circadian system (15, 27). *In vitro* results demonstrated that fetal SCN can develop a rhythm (28–30). This leads to the hypothesis that input from the maternal circadian system entrains fetal daily rhythms. Candidate maternal signal(s) or signals responsible for fetal circadian entrainment include melatonin, glucocorticoids, and dopamine (31).

## Maternal to Fetal Communication

Melatonin, dopamine, and corticosterone represent the three best-studied candidates for maternal-to-fetal communication of circadian rhythms. Each of these hormones has been tested for their capacity to synchronize fetal circadian rhythms *in vivo* or *in vitro*. None of these, however, are necessary for entrainment of the fetal circadian rhythm indicating there are parallel or compensatory pathways by which the mother can coordinate daily timing in her offspring.

## Melatonin

### Evidence for Rhythmic Expression in Humans and Rodents

The pineal gland secretes melatonin at night under control of a multi-synaptic pathway from the retina, SCN, and spinal cord (3, 32). Melatonin secretion depends on the environmental photoperiod, the length of time of light exposure (33). During the short days of winter, for example, more melatonin is secreted (33).

**Figure 1:**
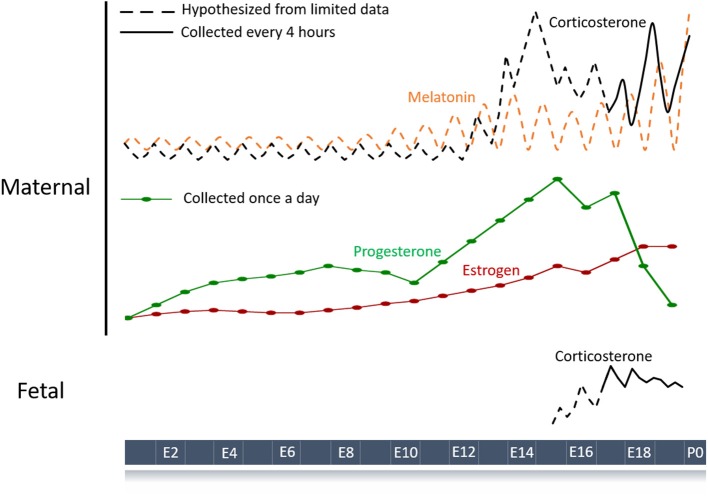
Schematic of hormone profiles over the course of the mouse pregnancy. Maternal hormones with a diurnal pattern are strong candidates for entraining fetal circadian rhythms and development. Hormone profiles throughout gestation have been measured (solid lines) or inferred (dashed lines) based on limited data (34–36). Melatonin and corticosterone are the major maternal hormones known to have circadian rhythms during pregnancy although other humoral signals like progesterone and estrogen could play a role. Fetal hormones such as corticosterone can also show circadian patterning and contribute to normal gestation.

Detailed studies on the rhythmic secretion of melatonin during pregnancy are limited (Figure 1). However, they do suggest that nighttime melatonin levels change during pregnancy. In pregnant rats, daytime levels of melatonin measured 7 h after daily lights on (Zeitgeber time 7, ZT7) remained the same as in non-pregnant rats, but nighttime (ZT19) melatonin levels gradually increased after embryonic day 12 (E12) and peaked right before parturition (34). Melatonin returned to non-pregnant levels by postpartum day 2 (34). In humans, there was a similar pattern in secretion. In one study, daytime melatonin levels did not change over pregnancy while nighttime serum melatonin increased significantly at 32 weeks compared to 24 weeks of pregnancy and returned to non-pregnant levels on postpartum day 2 (37). However, this study was limited to two daily measurements (at 2:00 a.m. and 2:00 p.m.) during the second and third trimester. In a separate study, daytime and nighttime serum melatonin were significantly higher in the third trimester compared to the first and second (19). The peak time of melatonin secretion did not change between the early pregnancy (9–13 weeks) and late pregnancy (24–37 weeks). (19). In both groups of women, serum melatonin exhibited diurnal variation with a peak between midnight and 4:00 a.m., consistent with the peak time found in non-pregnant women (19). This study did not follow the same women throughout their pregnancy; instead, they utilized samples from different women who were either in their first, second, or third trimester. Future work should track how daily profiles of melatonin change their level and timing in individual term and pre-term pregnancies.

### Evidence for Maternal-to-Fetal Transfer

When melatonin is administered to rats and humans during pregnancy, it passes through the placenta into the fetal circulation unaltered (38, 39). In E21 pregnant rats, ~75% of radioactive [^125^I]iodo-melatonin was found in fetal cardiac blood six minutes after tail vein injection (39). In humans, similar levels of serum melatonin occur in the maternal umbilical circulation (38). Administration of melatonin to term women led to a comparable increase in serum melatonin levels in the maternal and umbilical circulation (38). These results indicate that maternal melatonin can reach the fetus although when and how quickly remain unknown.

### Evidence for Fetal Expression of the Receptor(s)

Melatonin receptors are widespread in fetal tissues and the placenta (40, 41). Melatonin begins binding to receptors in the fetal rat SCN on E18, 1 day after SCN neurogenesis (41). In humans, melatonin binding in the SCN occurs at 18–19 weeks of gestation (42). However, it has not been determined whether binding occurs prior to 18 weeks.

### Evidence Hormone Is Necessary/Sufficient to Entrain Fetus

Daily rhythms in maternal melatonin likely act as an indicator of the maternal circadian time and entrain the fetus to the environment (41). Exogenous administration of melatonin to SCN-lesioned pregnant hamsters caused their pups to have synchronized behavioral rhythms after birth depending on the time of day of the injections *in utero* (43). Maternal pinealectomy of rats early in gestation caused the neonatal pups to be less synchronized in their drinking activity compared to control mice (44). Injections of exogenous melatonin to pinealectomized rats late in gestation was sufficient to synchronize the drinking rhythms of the pups (44).

However, entrainment of the fetus to the maternal schedule does not require melatonin (45, 46). Mouse strains which lack melatonin production still show daily rhythms in physiology and behavior that appear synchronized among the offspring. Furthermore, pinealectomy of rat dams failed to prevent entrainment of fetuses to the maternal daily schedule (47).

In summary, daily melatonin levels increase in amplitude, but do not appear to change their phase, over the course of pregnancy. Melatonin readily crosses the placenta to enter the fetal circulation where melatonin receptors are found prior to birth. It suffices to entrain the fetus when administered exogenously at different times of day, but it is not necessary for entrainment, suggesting it acts as a redundant signal to entrain the fetus. Future work should help us to understand how rhythmic expression of melatonin may change and impact fetal daily rhythms over the course of a healthy pregnancy in women.

## Dopamine

### Evidence for Rhythmic Expression in Humans and Rodents

Early studies using *in utero* injections also implicated dopamine as maternal signal to time fetal circadian rhythms. Daily expression of dopamine during pregnancy has not been studied in either humans or rodents, but two studies in pregnant rhesus macaques provided some evidence for circadian regulation (48, 49). In one study, pregnant rhesus macaques had blood collected every three hours from 127 to 135 days of gestation (term = 167 days) (48). Maternal plasma and amniotic fluid dopamine levels did not exhibit significant changes with time of day (48). In the second study which collected maternal plasma from 129 to 154 days of gestation, dopamine levels were higher during the morning (0900 and 1,200) than at night (2,400, 0300, and 0600) (49). In humans, there are no studies examining diurnal variation of plasma dopamine in women, but there has been one study on men. Plasma dopamine levels in men peaked around waking (~8:00 a.m.) and were at their nadir in the middle of sleep (~3:00 a.m.) (21). Because prolactin, estrogen, and dopamine have an antagonistic relationship in the brain with dopamine levels rising when prolactin levels decrease and vice versa (50), it will be important to look for sex differences in daily dopamine profiles. Considering circulating levels of prolactin and estrogen change during menstruation and pregnancy (51), future work should also compare daily dopamine levels in non-pregnant and pregnant women.

### Evidence for Maternal-to-Fetal Transfer

Dopamine may cross the placenta and enter the fetal circulatory system. Women showed an increase in dopamine concentration in the amniotic fluid between the second (15–20 weeks of gestation) and third trimester (36–41 weeks), but not in maternal or fetal plasma (52). The amniotic fluid concentration had a 15-fold increase and was significantly higher than fetal and maternal plasma levels (52).

In pregnant rats, dopamine levels in fetal plasma decreased, in amniotic fluid increased while maternal plasma levels did not change from E18-E22 (53, 54). Some of the increase in dopamine levels in the amniotic fluid is likely from conversion of fetal L-dopa, but it is not understood what proportions of the amniotic fluid dopamine levels are contributed by the mother and fetus (55). In E19 pregnant rats injected with radioactive dopamine, the fetal plasma had half the concentration found in the maternal plasma 1 h after the injection (56).

### Evidence for Fetal Expression of the Receptor(s)

D_1_ dopamine receptors are widely found in the fetal rat brain at E20 (57). At E18, D_1_-dopamine receptors are found in the SCN (58). Injections on E19 with the D_1_ agonist SKF 38393 caused *c-fos-* induction in the fetal SCN. In humans, D_1_ receptors were found in the SCN at 22 weeks of gestation (59). However, it is unknown whether expression occurs prior to 22 weeks and how it may change at different stages of pregnancy.

### Evidence Hormone Is Necessary/Sufficient to Entrain Fetus

Dopamine can affect fetal circadian timing. One injection given on E15 (birth occurs on E16) to SCN-lesioned hamsters produced pups with daily locomotor activity starting approximately 12-h apart depending on whether the mother received melatonin or dopamine (60). Melatonin and dopamine are known to act antagonistically in the chick retina by modulating intracellular cAMP levels (61).

In summary, dopamine, like melatonin, could fulfill the criteria as circadian entraining signals. Dopamine levels vary with time of day during pregnancy in some studies, but we need better measurements in women. In rodents, dopamine crosses the placenta to enter the fetal circulation where D_1_ receptors are found prior to birth and exogenous dopamine can shift fetal rhythms in anti-phase to melatonin, but there remains much we do not know about when and how endogenous dopamine may entrain the fetus.

## Corticosterone

### Evidence for Rhythmic Expression in Humans and Rodents

Glucocorticoids, such as corticosterone, are released in response to stress, but also exhibit a circadian rhythm in their release (62). Serum corticosterone peaks just prior to activity onset, driven by the SCN (62). SCN lesioning abolishes corticosterone rhythms (63).

There is limited data on the rhythmic expression of glucocorticoids over the course of pregnancy. In humans, serum cortisol peaked a few hours after waking throughout pregnancy with the morning peak progressively increasing between weeks 11 and 22 and then remaining high until initiation of labor (23). A second study found similar daily cortisol profiles from weeks 30–39 of pregnancy (24). Mice also showed an increase in the morning levels of maternal serum corticosterone starting around E11-E14, but with the peak occurring near the beginning of the night, their locomotor active phase (35).

### Evidence for Maternal-to-Fetal Transfer

Corticosterone and other glucocorticoids are important to the maturation of several fetal organs (64, 65). In mice, fetal serum corticosterone levels are a product of maternal corticosterone that has crossed the placental barrier and corticosterone produced from the fetal adrenal cortex (66). Knocking out the corticotropin-releasing hormone (CRH) gene (*Crh*) in the dam and the fetuses, caused the fetuses to die following birth due to underdeveloped lungs (67). However, when the mother is heterozygous (*Crh*^+/−^*)* for the *Crh* gene while the fetuses are knockouts (*Crh*^−/−^*)*, the fetuses exhibited normal development of their lungs through placental transport of maternal corticosterone (67). *Crh*^+/−^ dams with *Crh*^−/−^ fetuses did exhibit day-night differences in fetal corticosterone at E16.5, suggesting that maternal-derived corticosterone crosses the placenta in a rhythmic manner (66). The placenta regulates glucocorticoid passage from the mother to the fetus through 11-β hydroxysteroid dehydrogenase type 2 (11βHSD2) (68). 11βHSD2 metabolizes most of the maternal glucocorticoids, preventing them from crossing the placenta and entering the fetal blood (68). Thus, the daily rhythm in maternal corticosterone release, placental transport, and metabolism by 11βHSD2 appears to produce peak fetal corticosterone levels at night (66). It will be intriguing to learn whether the presence of, or daily rhythm in, glucocorticoids is critical for fetal development.

### Evidence for Fetal Expression of the Receptor(s)

Although there are no studies on the expression of glucocorticoid receptors in the human fetal brain, rats and mice express the glucocorticoid receptor (*rNr3c1*) in the fetal SCN where it exhibited time of day differences in mRNA expression (69). This contrasts with multiple reports which found no glucocorticoid receptors in the adult SCN, but widespread expression in other tissues (70, 71). This may indicate a special role for maternal glucocorticoid control of fetal circadian rhythms during pregnancy.

### Evidence Hormone Is Necessary/Sufficient to Entrain Fetus

Glucocorticoids may act as an entraining agent for the fetus. *In vitro* work showed that chronic exposure to a glucocorticoid receptor agonist, dexamethasone, lengthened the circadian period, and acute exposure shifted the time of peak PER2 expression depending on the time of day that it was added to fetal mouse SCN explants (69). Unlike dopamine injections during pregnancy, dexamethasone injections cause an acute reduction in *c-fos* expression levels in the fetal SCN (69).

In summary, if, when and how glucocorticoids entrain fetal circadian rhythms remains unknown, but they are well-positioned to play an important role. Glucocorticoid levels increase in amplitude during the first two-thirds of pregnancy, with no apparent change in the time of their daily peak. They readily cross the placenta to enter the fetal circulation where glucocorticoid receptors are found prior to birth and, when administered to fetal tissue, they can shift circadian rhythms.

## Other Signals that Might Influence Maternal-Fetal Circadian Communication

To complete this review of potential daily signals from the mother that could impact fetal circadian rhythms and development, we include three additional signals that, while understudied, have shown promise in fulfilling the requirements as circadian coupling factors: progesterone, estrogen, and maternal feeding.

### Progesterone and Estrogen

#### Evidence for Rhythmic Expression in Humans and Rodents

Although serum progesterone and estradiol concentrations have been well characterized across gestation in women and rodents (36, 72, 73), little is known about the diurnal variation of these hormones before or during pregnancy. In humans, progesterone and estrogen levels progressively increase throughout pregnancy (74, 75). One study examined 5 women for diurnal variations in progesterone at 6–10 weeks of gestation and concluded that there was no diurnal variation (25). In a study examining 17 non-pregnant women at different stages of the menstrual cycle, women in the follicular phase displayed diurnal changes in hormone levels with progesterone peaking in the early morning (~10:00 a.m.) and estradiol peaking around 1:00 a.m. Women in the luteal phase did not display diurnal variation in progesterone or estradiol levels (18). However, two other studies of women found diurnal profiles in progesterone levels during the luteal phase with variations in the timing of peak between the women (76) and diurnal profiles of free estradiol throughout their menstrual cycles (77). In pregnant women, serum and urine estradiol concentration in the third trimester exhibited time of day differences, peaking around noon (24, 78).

In mice, progesterone levels steadily rise over the course of pregnancy before declining prior to labor (73). Around E18, an increase in 20α-hydroxysteroid dehydrogenase facilitates the progesterone withdrawal prior to the initiation of parturition (79). One paper that examined serum progesterone levels over two 24-h periods of the rat pregnancy showed that progesterone levels have a peak around ZT9 and a nadir around ZT17 at both E15 and E21 (80).

To fully explore the potential of maternal and placental hormones in circadian signaling to the fetus, we need more information about their receptor distributions in fetal tissues, their ability to cross the placenta at different times of day, their necessity for circadian gating of birth and fetal circadian rhythms, and their effects on each other. For example, estrogen and progesterone may modulate fetal glucocorticoid levels. In human placental extracts, estrogen, and progesterone reduced 11βHSD2 expression which may allow increased transport of glucocorticoids from the mother to the fetus (24).

The placenta has an important role as an endocrine organ, producing hormones that are released into the fetal and maternal circulation (81). The placenta produces progesterone and estrogen and also transports maternally derived hormones into the fetal circulation (81). Some hormones pass through the placenta unchanged, such as melatonin, while others are metabolized into inactive forms, such as glucocorticoids (81). Some placental hormones (e.g., lactogen, growth hormone, and human chorionic gonadotropin) have not been found to exhibit diurnal variations during the latter half of pregnancy (82–84), and thus, would seem less likely to serve as circadian entrainment factors. Because the sources, timing and levels of daily hormone secretion can change throughout pregnancy, we must continue to consider their convergent actions on the proximal factors that entrain fetal circadian rhythms.

#### Maternal Feeding

Finally, hormonal rhythms are not the only possible entraining signals. Maternal feeding can act as an entraining signal in certain circumstances (16, 85, 86). For example, offspring from SCN-lesioned dams drank at random times of the day relative to each other, but showed inter- and intra-litter synchronization following 4-h restricted daily feeding during pregnancy (16). The restricted feeding reestablished rhythms in locomotor activity and body temperature in the dam which may have directly, or indirectly, had a role in entraining the pups. In another experiment, when pregnant rats in a light cycle shifted their locomotor activity to anticipate 4 h of daytime food availability (ZT5-9), the E22 fetal SCN explants showed a similar shift in daily *Per1-luc* expression (85). Thus, maternal feeding schedules can impact fetal circadian timing, perhaps through humoral or other (e.g., metabolic) pathways. For example, because restricted feeding in non-pregnant SCN lesioned mice can restore daily rhythmicity in corticosterone (87), maternal feeding could act through glucocorticoid signaling to entrain fetal rhythms. How and when daily maternal feeding-related signals synchronize fetal circadian rhythms remains to be studied.

## Conclusion

Circadian rhythms are important in pregnancy. When development of the fetal circadian system is disturbed, the offspring are at an increased risk for cardiovascular disease, obesity, and other health problems (88, 89). Yet, we know little about how the fetus entrains to the mother and how this might impact gestation length. Melatonin, dopamine, and glucocorticoids fulfill many of the criteria for factors that synchronize the offspring. Future studies should elucidate how these hormones change during pregnancy in women. Less invasive methods to measure circadian hormones with high temporal resolution could help us identify pregnancies that are at risk for complications. Additionally, some of these hormones are exogenously administered during pregnancy, but without consideration of their normal diurnal variation. For example, women at risk of preterm birth receive antenatal corticosteroids as part of standard care to improve neonatal outcomes (90). Knowing the appropriate daily profile and roles of glucocorticoids (e.g., in entraining the fetus) could inform the best times of day and doses for steroid treatment to promote fetal development and circadian entrainment. Daily rhythms of maternal and placental signals and their roles in entrainment and birth timing is an area ripe for study and with tremendous potential for translation into clinical care for healthy pregnancies.

## Author Contributions

All authors listed have made a substantial, direct and intellectual contribution to the work, and approved it for publication.

### Conflict of Interest

The authors declare that the research was conducted in the absence of any commercial or financial relationships that could be construed as a potential conflict of interest.
